# How to build phylogenetic species trees with OMA

**DOI:** 10.12688/f1000research.23790.1

**Published:** 2020-06-04

**Authors:** David Dylus, Yannis Nevers, Adrian M. Altenhoff, Antoine Gürtler, Christophe Dessimoz, Natasha M. Glover

**Affiliations:** 1 Swiss Institute of Bioinformatics, Lausanne, 1015, Switzerland; 2 Department of Computational Biology, University of Lausanne, Lausanne, 1015, Switzerland; 3 Center for Integrative Genomics, University of Lausanne, Lausanne, 1015, Switzerland; 4 Department of Computer Science, ETH Zurich, Zurich, 8092, Switzerland; 5 Department of Genetics, Evolution and Environment, University College London, London, WC1E 6BT, UK; 6 Department of Computer Science, University College London, London, WC1E 6BT, UK

**Keywords:** phylogenetics, phylogenomics, species tree, OMA, Orthologous Matrix

## Abstract

Knowledge of species phylogeny is critical to many fields of biology. In an era of genome data availability, the most common way to make a phylogenetic species tree is by using multiple protein-coding genes, conserved in multiple species. This methodology is composed of several steps: orthology inference, multiple sequence alignment and inference of the phylogeny with dedicated tools. This can be a difficult task, and orthology inference, in particular, is usually computationally intensive and error prone if done *ad hoc*. This tutorial provides protocols to make use of OMA Orthologous Groups, a set of genes all orthologous to each other, to infer a phylogenetic species tree. It is designed to be user-friendly and computationally inexpensive, by providing two options: (1) Using only precomputed groups with species available on the OMA Browser, or (2) Computing orthologs using OMA Standalone for additional species, with the option of using precomputed orthology relations for those present in OMA. A protocol for downstream analyses is provided as well, including creating a supermatrix, tree inference, and visualization. All protocols use publicly available software, and we provide scripts and code snippets to facilitate data handling. The protocols are accompanied with practical examples.

## Introduction

Inferring accurate and complete species phylogenies is a fundamental problem in biology ^1^. Traditionally, species trees have been inferred using ubiquitous marker genes such as 16S rRNA ribosomal genes ^2^. However, as there are fewer sites to sample, using only one gene per species limits the resolution of the inference and the phylogeny of the gene does not necessarily reflect the evolutionary history of the entire species. For this reason, species phylogenies are now overwhelmingly inferred from multiple genes ^3^. As long as one takes the necessary precautions, notably selecting true orthologs for their comparisons, (see 3 for common pitfalls in phylogenomics), multilocus phylogenies are better resolved and more robust ^4^. Recently, multiple protein-coding genes were used to infer a tree comprising ~3000 species, but this was still limited to a small number of concatenated ribosomal genes ^5^. With the rise of next-generation sequencing, many hundreds of genes can now be considered when building species trees, which tremendously increases the available information for the inference.

To make use of all available genes, one needs to identify groups of genes that emerged from a common ancestral gene solely through speciation. These sets of genes, in which all pairs of genes are orthologs ^6^, are commonly referred to as Orthologous Groups (OGs). The inference of OGs is non-trivial due to additional evolutionary events such as gene duplications, gene losses or horizontal gene transfers ^7^. Furthermore, there are numerous algorithms for inferring orthology which can result in different OG composition, further complicating matters. In the Orthologous MAtrix (OMA), OGs can have at most one representative gene per species. This type of OG is provided by a few orthology databases such as BUSCO ^8^ and OMA ^9, 10^.

In this tutorial, we focus on OGs obtained from OMA. Alternatively called “OMA Groups,” they are specifically designed for species tree inference. OMA Groups are defined as gene families that contain genes which are all orthologous to each other, with a maximum of one gene per species ^11^. These are stringently computed orthologs that make use of all the available species in OMA. Another term used for this type of groups are marker genes, or phylogenetic marker genes. Moreover, OMA Groups have repeatedly been shown to produce reliable trees ^10^ and its underlying algorithm was shown to accurately infer OGs in a large-scale benchmark ^12^.

The OMA algorithm is freely available as an open source software tool (OMA Standalone ^13^) and integrates well with the public OMA Browser ( https://omabrowser.org), a database that provides orthology information among more than 2300 genomes across the tree of life. Here, we show how to leverage the publicly available orthology data to infer phylogenetic species trees.

First, we set up the prerequisites and explain how to search for pre-computed OGs for species of interest in the OMA database. Then, we show how to infer a species tree under two scenarios: (1) using only species that are present in the public OMA database, or (2) using species in OMA in addition to other genomes not available in the database, e.g. data obtained from sequencing the genome of a new species. Finally, we show how to do downstream processing and tree inference. Each of these steps is illustrated by an example on real data.

## Materials

The tools needed for this tutorial can be found in Table 1. All commands can be run from the command line and/or with python scripts. We reference four tree inference software tools, but there are many other alternatives (see List of phylogenetics software).

**Table 1.** Computational tools needed for making a phylogenetic species tree using OMA. Note that four phylogenetic tree inference softwares are given, but only one (user preference) is needed to complete this tutorial.

Tool	Use case	How to get it
Command line	Mandatory to run the commands written in this tutorial	Installed by default on Unix and Mac
Python 3	Python language interpreter. Mandatory for running the scripts used in this paper	https://www.python.org/downloads/
OMA Browser	Needed to import orthology relations used for tree inference	https://omabrowser.org
OMA standalone	Needed to infer orthology for data not available in the OMA Browser	https://omabrowser.org/standalone/#downloads
MAFFT	Multiple sequence alignment software	https://mafft.cbrc.jp/alignment/software/
High Performance Computing (HPC)	Needed if a high amount of computation is involved	Institutional infrastructure
IQTree	Phylogenetic tree software	http://www.iqtree.org/#download
RaXML	Phylogenetic tree software	https://cme.h-its.org/exelixis/web/software/raxml/index.html
Phylobayes	Phylogenetic tree software	http://megasun.bch.umontreal.ca/People/lartillot/www/download.html
PhyML	Phylogenetic tree software	http://www.atgc-montpellier.fr/phyml/
Phylo.io	Phylogenetic tree visualization website	http://phylo.io

Two examples will be used in this tutorial, one to illustrate Protocol 1, and another to illustrate Protocol 2. Both examples can be downloaded from Figshare ^14^. Instructions on how to obtain the data from the OMA Browser for Protocol 1 is described in the next section, so it is not required to download to complete this tutorial. However, Protocol 2 is used to add external genomes. For this example we chose two genomes available in the OMA Browser, but we set them aside after downloading the data. We then re-add them as external data (FOMPI.fa and YEAST.fa). For reproducibility, these proteomes can be found in the data/AddedGenomes subdirectory of Protocol_2. The rest of the data included in the example tarball are OGs and alignment files needed to compute the trees, which are also included as results.

The tree computations on these data have been performed using both RaXML 8.2.12 and IQTree 1.7.beta17, as specified in the PDF accompanying the examples.

## Protocols

Phylogenetic tree inference using OMA is done in three steps: getting OG data, aligning all sequences of every OG and combining them into a supermatrix, and finally, using tree inference tools on the supermatrix. Depending on the species requirement, two options are available to obtain OG data, they are detailed in subsection Protocol 1 and 2. Protocol 1 is the fastest and can be used if all species of interest are available on the OMA Browser. Alternatively, Protocol 2 is for the cases when new genomes must be added, or when solely using data computed by OMA Standalone. Later steps are the same for both cases and are addressed in Protocol 3.

### Protocol 1: Export marker genes to make a phylogeny of species found in OMA

This method is the quickest way to obtain data to build a phylogenetic species tree, but is only useful if one is interested in making a tree from species already in the OMA database. To do this, the *Export marker genes* function in the browser takes advantage of the precomputed OMA Groups. As mentioned in the Introduction, OMA Groups are a specific type of OG which contain sets of genes that are all orthologous to one another. This implies that there is at most one gene from each species in a group.

***Finding species of interest in OMA.*** The OMA public database and all related information are accessed through the OMA browser ( https://omabrowser.org/). One can search for species of interest in the OMA database by browsing through the available data in OMA using the release info page (in the upper left of the home screen: *Explore -> Release information*). Two browsing options are available, the default one is through an interactive tree, with colours indicating domains of life: bacteria are blue, archaea are green and eukaryotes are red. The other option is a table viewer featuring a search bar and accessed through the *View as Species List* icon in the top right of the table.

**Figure 1.**
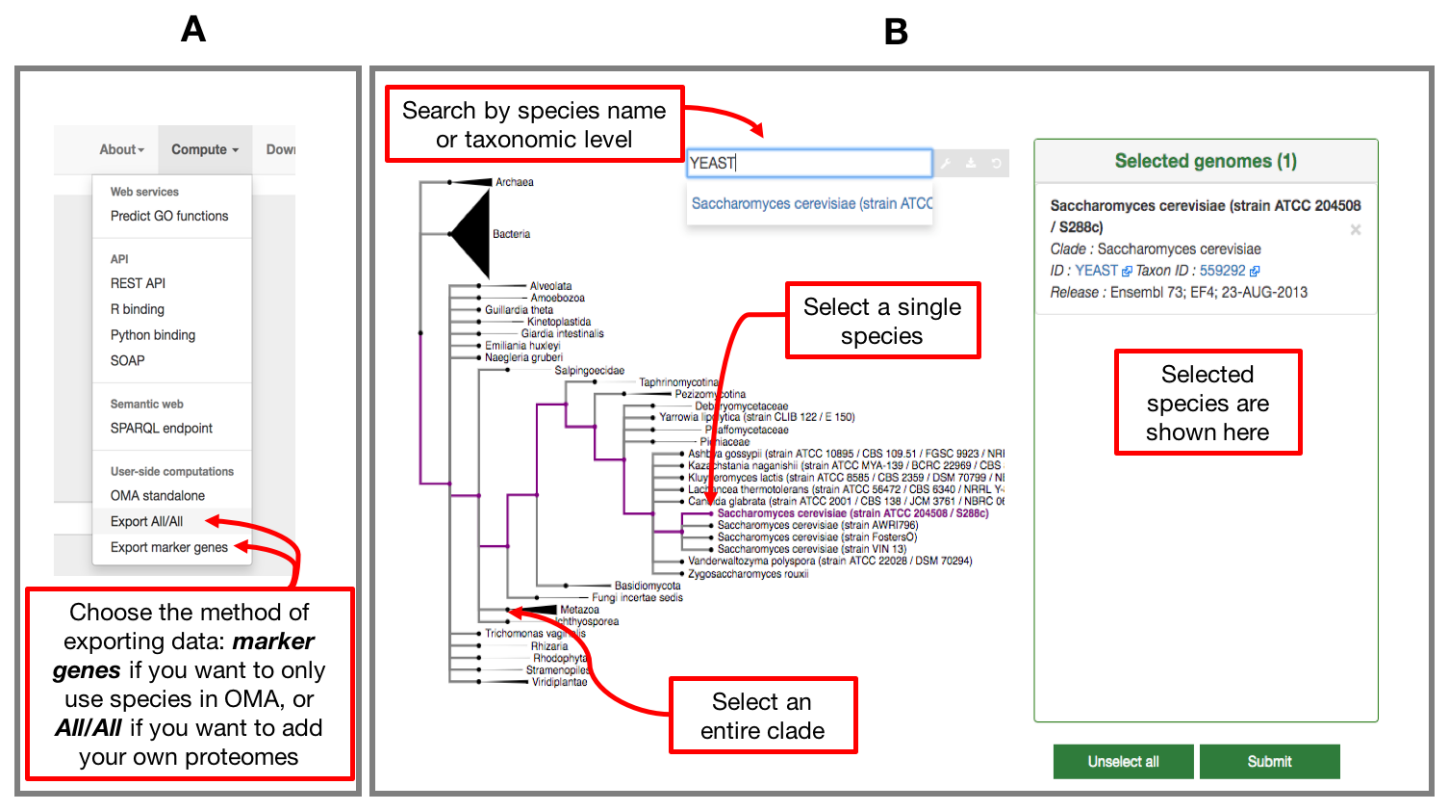
Exporting data from OMA for building a species tree. **A**) Choose which type of data to export from the *Compute* tab on the right hand side of the home page. **B**) Select your genomes from those in the OMA database by using the interactive species tree, based on the NCBI taxonomy.

***Export the relevant data from OMA.*** The way to obtain OGs with only species present in the OMA database is by using the *Compute -> Export marker genes* option ( Figure 1A). This will open a page which allows the user to select species. Species can be searched by name or clade. A whole clade can be selected by clicking on the node ( *select all species*). A single species can be selected by clicking on the leaf ( *select species*). All selected species will be displayed in the right box with additional species information (release info, taxon id, etc.) ( Figure 1B).

***Specifying the Minimum Species Coverage and Maximum Number of Markers parameters.*** After species selection, exported OGs will depend on the minimum fraction of covered species and the maximum number of markers parameters:

- *Minimum species coverage:* the lowest acceptable proportion of selected species that are present in any given OG in order to be exported.
  A more permissive (lower) minimum species coverage will result in a higher number of exported groups. Choosing this parameter depends on the number of and how closely related are the selected species. For instance, consider the *Drosophila* clade versus chordates clade (20 and 116 species in current release, respectively). If one selects the 20 *Drosophila* genomes and sets the minimum species coverage to 0.5, only OGs with at least 10 *Drosophila* species will be exported. In the current release, this results in 11,855 OGs which meet this criteria. If using the same 0.5 minimum species coverage for the chordates, it results in 14,357 OGs exported. On the other hand, for a 0.8 minimum species coverage, 7,886 and 6,329 OGs are exported for *Drosophila* and chordates clades, respectively.
- *Maximum number of markers:* the maximum number of OGs/marker genes to return. To consider as much information as possible in the tree inference, remove any limit by setting this parameter to -1, in which case all OGs fulfilling the minimum species coverage parameter will be returned. To speed up the tree inference, set this value to below 1000 genes.

After filling in the parameters and submitting the request, the browser will return a compressed archive (“tarball”) that contains a fasta file with unaligned sequences for each OG. Depending on the size of the request, it may take a few minutes for this operation to complete.

As an example for Protocol 1, we performed an analysis on 20 yeast species, using only OGs shared by all species ( *Minimum species coverage*: 1) and no limit to the number of OGs retrieved ( *Maximum number of markers*: -1). We obtained 169 OGs with this query. The corresponding data can be found on Figshare ^14^, in the Protocol_1 folder.

Upon exporting the marker genes, i.e. OGs, from OMA, the data can be used to make a phylogenetic species tree (skip to Protocol 3: Downstream processing and tree inference).

### Protocol 2: Export precomputed OMA all-against-all data as a backbone to add your own genomes and use with OMA standalone

Orthology computation first starts with an all-against-all alignment phase一comparing all proteins in every species of interest to each other. If genomes to be included in the species phylogeny are not present in OMA (hereafter referred to as “added genomes”), it is necessary to first compute orthology predictions for the combined set of species (those in OMA plus the added genomes). This approach is computationally more expensive and requires that computations are performed on a local machine or high performance computing cluster (HPC). However, by using the OMA Browser’s *Export -> All/All* option, one can take advantage of the precomputed all-against-all data for those species in OMA, saving time. The following protocol describes how to make use of this data and run OMA Standalone, the software for running the OMA algorithm on added genomes. In the case where the user wants to only use genomes unavailable in OMA, skip to the “Running OMA Standalone” section.

***Export the all-against-all from OMA.*** Choose species which you want to combine with your own genomes by choosing *Compute -> Export All/All* from the home page ( Figure 1A). This will lead to an interactive species tree of all the species in OMA, for which you can choose your species of interest to export ( Figure 1B).

**Figure 2.**
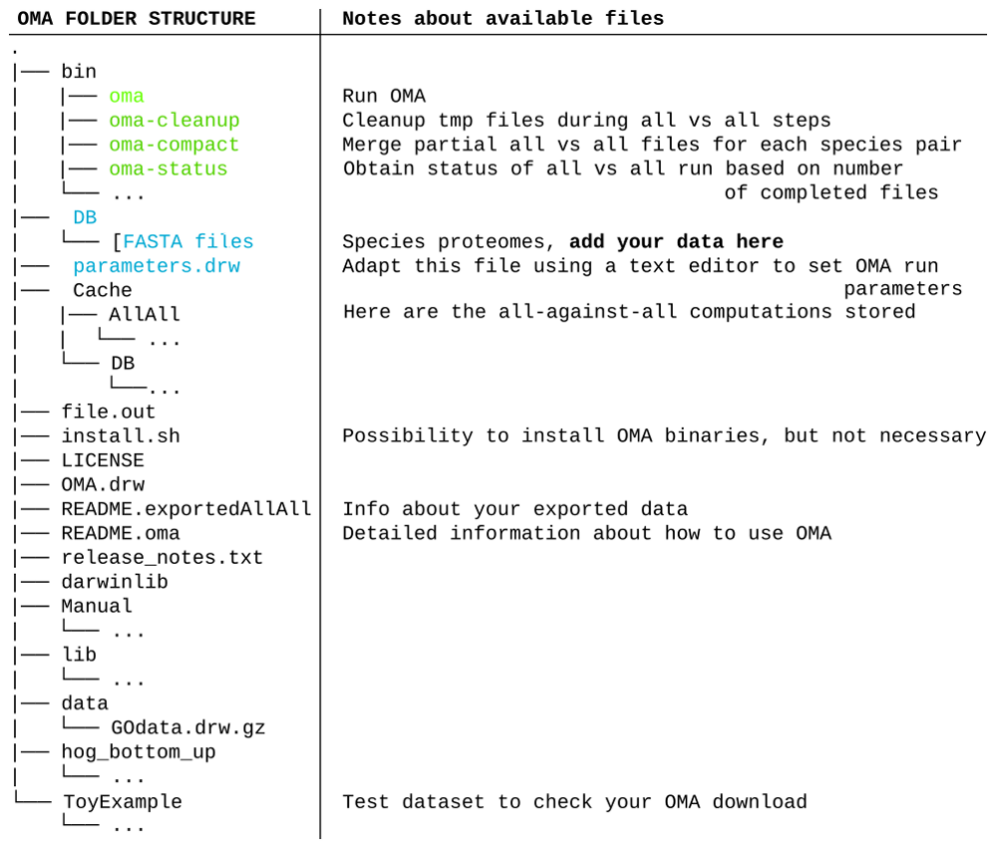
Tree organization of the tarball downloaded through the OMA Browser after exporting an all-against-all of selected species. The important files and folders are coloured. In green are the executable files mentioned in the course of the tutorial. In blue are the files and folder that will need to be modified. Other files and folders (in black) will not be used in the course of the tutorial. Files and folders not shown are represented by three dots.

After selecting species and clicking submit, the OMA Browser will export a tarball (described in Figure 2) which contains:

- The all-against-all alignments of the selected species, found in the folder “Cache.”
- All exported genomes, in the format of protein fasta files, found in the folder “DB.”
- The full OMA standalone software tool. No need to download it separately.

***Combining the added genomes with exported OMA data.*** Next, the added genomes data must be combined to the OMA data. For this procedure, the added genomes data must fulfil certain conditions:

- Each genome is in the form of a fasta file, containing *protein* sequences.
- The name of the fasta file is either the species name or the 5-letter UniProt identifier of the added species (e.g. ARATH for *Arabidopsis thaliana*).
- Each sequence in the fasta file has a clear and unique identifier. There must be no square brackets or other special characters.

If these conditions are fulfilled, these fasta files must be put into the DB folder with the other exported OMA genomes ( Figure 2), where they will be considered as a unique dataset for the following steps.

***Setting the parameters for OMA standalone.*** Before starting the computation, it is wise to adjust the parameters file, called “parameters.drw” ( Figure 2), which can be edited with any text editor. If the goal is to only generate a dataset for species phylogeny inference (and not keeping other unrelated orthology inferences), one can avoid doing computations and generating output files that are not needed by the following:

- Uncomment (remove the # from) all the lines starting with WriteOutput EXCEPT #WriteOutput_OrthologousGroupsFasta := false. By keeping that one commented, OMA standalone will produce one fasta file for each inferred OG.
- Deactivate the Hierarchical Orthologous Group inference, which is not needed here, by setting DoHierarchicalGroups := false;
- Likewise, deactivate the gene function prediction by setting DoGroupFunctionPrediction := false;
- *Tip:* do not omit a semicolon at the end of each uncommented statement.

***Running OMA standalone.*** To run OMA standalone, one needs to be aware that the OMA pipeline can be split into two parts: all-against-all homology inference and orthology calling. Because OMA can compute Smith-Waterman alignments in parallel for all species which were not exported from OMA (see Export the all-against-all from OMA), it is beneficial to perform the computations on a computer cluster. However, if the dataset is small (e.g. 2–3 additional genomes), the computations can be run locally on a standard computer.

To run OMA standalone on a small dataset locally:

1. Within the extracted tarball folder you can start the computation with the command line:
$: bin/oma -n NR_PROCESSES
  NR_PROCESSES should not be higher than the number of CPUs you have available on your machine.

For a larger dataset, we recommend the use of an HPC cluster. We recommend breaking up the computations into two parts: first the all-against-all part, then the orthology inference part:

1. Create a submission script for your cluster. Examples of submission scripts are provided at https://omabrowser.org/standalone/#schedulers and https://lab.dessimoz.org/blog/media/2020/04/omastandalone_cheat_sheet.pdf.
2. Make sure that the submission script enters the folder into which the tarball was extracted, by either running the script from inside that directory or using the cd command appropriately.
3. The line to start the OMA all-against-all computation in the submission scripts is:
$: bin/oma -s
  The -s option means stop after the all-against-all phase. Since this part can be parallelized, we recommend using job-arrays. For this you need to set the number of parallel jobs as an environment variable (export NR_PROCESSES=100) and use the job-array syntax in the submission script (e.g. in LSF: bsub -J oma[1-$NR_PROCESSES] bin/oma -s). For environments with limited runtimes/walltimes see https://omabrowser.org/standalone/#advanced%20optimisations.
4. Check whether the all-against-all computation is finished using:
$: bin/oma-status -i
  This command will output a file formatted as:
Summary of OMA standalone All-vs-All computations:
--------------------------------------------------
Nr chunks started:  A  (D.D%)
Nr chunks finished: B  (E.E%)
Nr chunks finished w/o exported genomes: C (F.F%)
  Where the letters A, B, C, D, E and F represent numbers. Once the computations are completed, D should be equal to 0.0%, and both E and F to 100.0%
5. In the case where the jobs are finished but the all-against-all computation is still not complete, use the oma-cleanup and oma-compact commands before re-submitting.
$: bin/oma-cleanup
$: bin/oma-compact
  These commands remove partially finished output files in the Cache/AllAll folder and zip all partial computations that are finished to one file, respectively.
6. Once the all-against-all computation has finished, the final step is the orthology calling. This step is more memory intensive, requires a single process, and can be called with:
$: bin/oma

Once the computation finishes, all results will be stored in the newly-created “Output” folder. In this folder there will be an EstimatedSpeciesTree.nwk file that contains a phylogenetic tree that can be visualized using a tree visualisation tool such as Phylo.io ^15^. This is a distance tree based on the weighted average of the pairwise distances between sequences within the most complete OMA groups. This species tree is a rough estimate that is computed on the fly, and is not the final tree. It can be used as control to identify problems in the dataset but will not be as reliable as the tree inferred using the generated OGs later in this protocol. Therefore it is recommended to use the OGs to compute your own tree with external software. The OGs (OMA Groups) ^9^ can be found in the OrthologousGroupsFasta folder, with each OG containing at least two species.

Usually for the construction of phylogenetic trees, one would select only OGs that contain at least X% of species, as described above with the parameter *Minimum Species Coverage*. The python script filter_groups.py from the git repository associated to this publication ( http://doi.org/10.5281/zenodo.3786201 ^16^) can be used to filter the OMA groups that contain at least X MIN_NR_SPECIES (replace <MIN_NR_SPECIES> and <destination/directory> with your own values):
$: python filter_groups.py --min-nr-species <MIN_NR_SPECIES> --input Output/OrthologousGroupFasta/ --output <destination/directory>


For example, we performed an analysis adding two yeast proteomes hypothetically not available in OMA and 18 available yeast proteomes. As a first step, we downloaded the precomputed data for the 18 proteomes from the OMA Browser and launched the computation after adding two separate proteomes. Once the computation finished, we selected 880 OGs with at least 18 species as a dataset to construct a tree. The data used in this example is available from Figshare ^14^ in the Protocol_2 folder.

### Protocol 3: Downstream processing and tree inference

Once all selected OGs are obtained from either of the first two protocols, the next step is to align all sequences within each OG. This can be done with any Multiple Sequence Alignment (MSA) tools, in this example we use MAFFT ^17^. To run it, navigate to the folder containing the selected OGs and execute the following command, which runs mafft on each fasta file:
$: for i in $(ls -1 *.fa); do mafft --maxiterate 1000 --localpair $i > $i.aln; done


This command generates a MSA file (.aln) for each OG. In order to infer the phylogeny of the species from these alignments, they have to be concatenated in a single alignment commonly referred to as supermatrix. We provide a python script to automate this, concat_alignment.py, available on http://doi.org/10.5281/zenodo.3786201 ^16^. The --format-output option allows for choosing the output format of this concatenation, either fasta or phylip format (some phylogenetic software requires a specific format as input). Once the python script is downloaded or cloned, ensure that all alignments are in the same folder, and launch using the following command:
$: python concat_alignments.py <path>/<to>/<alignments>/*aln --format-output [fasta/phylip] > output


After computing the supermatrix, the phylogenetic tree can be inferred using any number of available software. We recommend choosing from the tools in Table 2, sorted by computing time and increasing precision.

**Table 2.** Recommended software and example commands for computing a phylogenetic tree. Parameters, such as memory or threads, may vary based on size of dataset.

Software for making phylogenetic trees	Example command
IQTree	iqtree -s alignment.phy -m LG -T 20 --mem 20G -seed 12345 -bb 1000
RaXML	raxml-ng --threads 20 --model LG+G8+F --seed 15826 --msa alignment.phy --all --bs-trees 100
PhyloBayes	pb_mpi -dc -gtr -cat -dgam 4 -x 10 1000 -d alignment.phy alignment.chain1

***Tree visualization.*** Most of the current phylogenetic inference tools provide trees in Newick format as output. In order to visualize such a tree, one can use the web-based viewer phylo.io ( http://phylo.io) or other tree visualization tools (e.g. Figtree, phylogeny.io, etc).

**Figure 3.**
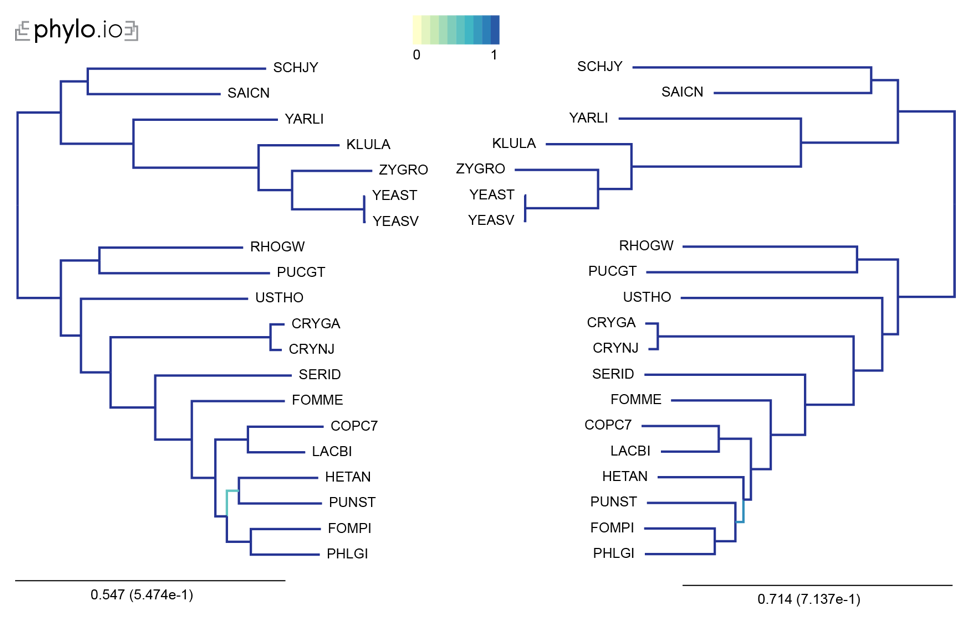
Comparison of phylogenetic trees computed by IQTree (left) and RaXML (right). Trees were computed with 20 Yeast species present in OMA. The leaves of the trees are the UniProt 5-letter species codes. The following export options were used: Minimum species coverage: 1, Maximum nr of markers: -1 (uncapped). 168 marker genes were exported. Visualization done with phylo.io; different shades of blue show variations in topology.

**Figure 4.**
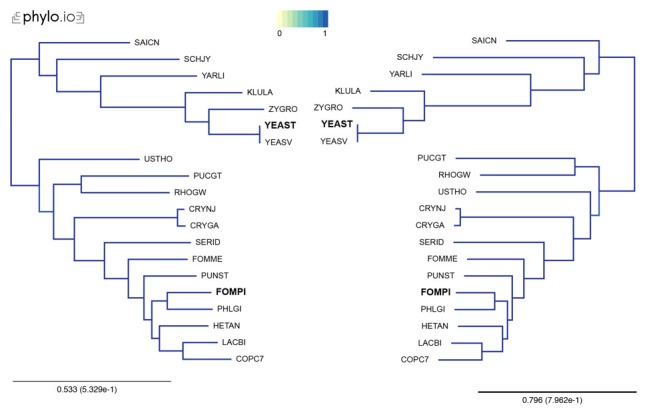
Comparison of phylogenetic trees computed by IQTree (left) and RaXML (right), using additional species. Trees were computed with 18 Yeast species present in OMA, plus two additional proteomes (YEAST and FOMPI). The leaves of the trees are the UniProt 5-letter species codes. Genes used to compute the tree had to be shared by at least 90% of the species (minimum species coverage: 0.9, maximum number markers: -1). This represents 880 OGs. Visualization done with phylo.io; different shades of blue show variations in topology (in this case both trees have identical topology).

In our examples, we inferred trees by aligning the sequences with MAFFT, concatenated the alignments using the aforementioned concat_alignments.py, and ran both IQTree and RaxML ( Figure 3 and Figure 4). The data used for and the results from the computations can be found on Figshare ^14^ (alignments in the *data/Alignments* folder, and trees in the *tree* folder). The exact code used for these examples is on http://doi.org/10.5281/zenodo.3786201 ^16^.

## Discussion and conclusion

With the wealth of genomic data available in an era of high-throughput sequencing, there is much to gain by making phylogenies from multiple gene families, rather than from one single gene. This can better represent the evolutionary history of a clade, because the evolutionary history of a single gene can be misrepresentative of a species evolutionary history. The principle of a supermatrix approach is that by combining multiple genes in one single phylogeny, one can combine the phylogenetic signal of multiple genes. One has to be careful however, to not combine “phylogenetic noise”. Orthologs selection is particularly important in this regard ^3, 18^, because errors in orthology inference could add genes that are not true orthologs, but rather paralogs descending from the ancestral genes by a duplication event. Thus, they would have a different evolutionary history than the sought species phylogeny.

OMA Groups (or Orthologous Groups) are a well suited set of orthologs for this kind of analysis, as the criteria used to compute these orthologs are stringent. They require that all genes are reciprocally closest genes in their respective species to all the other genes of the group and do not allow more than one gene in a species, thus excluding paralogs. In the *Quest for Orthologs* Benchmark ^12^, the community benchmark for orthology inference, OMA Groups are consistently the most specific inference, although lacking in recall. As potentially missing genes are less detrimental to phylogenetic determination than false predictions are ^19^, this is an appropriate choice of orthology inference method for this tutorial. Several phylogenies have already been published using OMA standalone or data from the OMA Browser, including those for archaea, sharks, spiders, worms, and insects, among others ^20– 24^.

This tutorial demonstrated how to carry out these different steps to infer a phylogenetic tree: orthology determination, sequence alignments, supermatrix construction, and phylogeny inference. It is designed to allow users to leverage the state of the art orthology inference provided by OMA Groups while reducing the necessary computation from their side, namely by relying on precomputed all-against-all alignments provided by the OMA Browser. We include code snippets and scripts that automate the whole process and ensure reproducibility of all phylogenetic analyses following this protocol. The tutorial is accompanied by practical examples with all data available on GitHub and Figshare.

For more information about the theory behind phylogenomics and the different methods, we refer the reader to recent reviews ^25– 27^. In the context of this tutorial, we used well-established MSA and phylogenetic tree inference tools. For the more difficult cases however, it advised to carefully choose which tool to use, including some tools which are not mentioned here. For more information about existing tools the readers are invited to turn to the relevant literature ^26, 28^. The protocols described here can be adapted to suit any other software compatible with standard data formats.

## Data availability

The imported OG data and the OMA standalone software can be obtained from the OMA Browser ( https://omabrowser.org), following instructions in this tutorial.

Figshare: Phylogenetic Tree Tutorial Example Data, https://doi.org/10.6084/m9.figshare.10780820.v4 ^14^.

Data are available under the terms of the Creative Commons Zero "No rights reserved" data waiver (CC0 1.0 Public domain dedication).

Additional python scripts (filter_groups.py and concat_alignments.py) are publicly available: https://github.com/DessimozLab/f1000_PhylogeneticTree

Archived scripts as at time of publication: http://doi.org/10.5281/zenodo.3786201 ^16^

License for scripts: MIT license

## Software availability

OMA Browser available at: https://omabrowser.org/.

Source code for OMA Standalone available from: https://github.com/DessimozLab/OmaStandalone/tree/v2.4.0

Archived source code of OMA StandAlone at time of publication: https://doi.org/10.5281/zenodo.3555595 ^13^.

OMA Browser license: Mozilla Public License version 2.
