# Supplementary material for: How to build phylogenetic species trees with OMA
Source: F1000Res. 2022 Feb 28;9:511. Originally published 2020 Jun 4. [Version 2] doi: 10.12688/f1000research.23790.2 (PMC9194518; doi:10.12688/f1000research.23790.2)
Supplement: Peer review history

# Peer review: Reviewer response for version 2

**DOI:** 10.5256/f1000research.121232.r125601

**Published:** 2022-06-13

**Authors:** Denis Baurain

**Affiliations:** 1 InBioS - PhytoSYSTEMS, Eukaryotic Phylogenomics, University of Liège, Liège, Belgium

Thank you for the revised version, which adequately addresses all my comments. I apologize for the delay in endorsing it.

Is the rationale for developing the new method (or application) clearly explained?

Yes

Is the description of the method technically sound?

Yes

Are the conclusions about the method and its performance adequately supported by the findings presented in the article?

Yes

If any results are presented, are all the source data underlying the results available to ensure full reproducibility?

Yes

Are sufficient details provided to allow replication of the method development and its use by others?

Partly

Reviewer Expertise:

Phylogenomics, comparative genomics, software engineering

I confirm that I have read this submission and believe that I have an appropriate level of expertise to confirm that it is of an acceptable scientific standard.

# Peer review: Reviewer response for version 2

**DOI:** 10.5256/f1000research.121232.r125602

**Published:** 2022-03-14

**Authors:** Jianbo Xie

**Affiliations:** 1 National Engineering Laboratory for Tree Breeding, College of Biological Sciences and Technology, Beijing Forestry University, Beijing, China

All my concerns have been solved.

Is the rationale for developing the new method (or application) clearly explained?

Yes

Is the description of the method technically sound?

Yes

Are the conclusions about the method and its performance adequately supported by the findings presented in the article?

Yes

If any results are presented, are all the source data underlying the results available to ensure full reproducibility?

Yes

Are sufficient details provided to allow replication of the method development and its use by others?

Yes

Reviewer Expertise:

Genetics; evolution

I confirm that I have read this submission and believe that I have an appropriate level of expertise to confirm that it is of an acceptable scientific standard.

# Peer review: Reviewer response for version 1

**DOI:** 10.5256/f1000research.26251.r84387

**Published:** 2021-06-02

**Authors:** Denis Baurain

**Affiliations:** 1 InBioS - PhytoSYSTEMS, Eukaryotic Phylogenomics, University of Liège, Liège, Belgium

This tutorial illustrates how to use the online OMA Browser and OMA Standalone package to assemble datasets of single-copy orthologous genes for phylogenomic inference. It covers two use cases: 1) all species of interest are present in the OMA Browser and 2) some species are not available and must thus be added by the user using OMA Standalone.

 Generally speaking, the structure and writing are clear, the figures and tables are appropriate, and the data and code underlying the examples are well described and made available in two public repositories (Figshare and Zenodo). Therefore, I do not have major comments. However, I think that it is possible to easily improve a number of minor points in the manuscript. I provide a list below, simply sorted by order of occurrence. There are no line numbers, so I try to provide some context when needed.

-  "16 rRNA ribosomal genes" should rather be "small subunit ribosomal RNA (SSU rRNA 16S/18S) gene" (no plural) to encompass the eukaryotic gene as well.
- "see 3 for common pitfalls in phylogenomics": Don't you mean Philippe *et al.* (2017) "Pitfalls in supermatrix phylogenomics ^1^" instead?
- "OGs can have at most one representative gene per species": You should probably say a word about recently diverged in-paralogs (which do not harm species phylogenies) because exactly single-copy genes barely exist.
- "among more than 2300 genomes across the tree of life": You might provide a few numbers here: bacteria, archaea, non-animals, non-plants - or at least recall the rationale for genome inclusion in OMA Browser because it is quite incomplete in terms of species diversity.
- "using species in OMA in addition to other genomes not available in the database": It is not clear if OMA Standalone requires conceptual translations of the sequences (i.e., proteins) or if it can translate DNA sequences on the fly; if it cannot, please specify that one needs protein sequences and not genomes to make use of OMA.
- "phylogenetic tree inference softwares": I am not a native English speaker, but "software" is uncountable; maybe you mean "software packages"?
- IQtree, RaxML, Phylobayes, Figtree etc: Please try to use the correct mixture of uppercase and lowercase letters in each name across the manuscript: IQ-TREE, RAxML, PhyloBayes, FigTree.
- A better download address for PhyloBayes would be: http://www.atgc-montpellier.fr/phylobayes/.
- The interface of OMA Browser has slightly changed since the manuscript has been written. Two details that would need updating: "in the upper left of the home screen" is incorrect [for "Explore"] and "Compute" has now become "Download".
- "To speed up the tree inference, set this value to below 1000 genes": One wonders how these <1000 genes would be prioritized and selected; this should be mentioned here.
- "The name of the fasta file is either the species name or the 5-letter UniProt identifier...": This is not specific enough; what about whitespace, dots in strain names, etc? Are they allowed? Similarly, you should better define "other special characters" just below.
- "...and not keeping other unrelated orthology inferences": This is a bit mysterious. I am aware that OMA can do more than what you demonstrate here, but this sentence should probably be slightly expanded with some examples.
- "...use the job-array syntax in the submission script": This is the part where reproducibility is not straightforward. In particular, is there a master OMA instance controlling the parallelization or is this left to the user? From the code snippet provided, it is not clear, albeit the output of the oma-status command suggests that there is some orchestrator freeing the user from this chore. Please explain a bit more.
- "we selected 880 OGs with at least 18 species as a dataset": Considering Figure 4, I understand your point: 90% of 20 species is 18. However, since you start with 18 genomes and add two "new" ones, the re-use of the number "18" is confusing to the user. As the analyses are done, I would not suggest to change this number, but rather to explain directly the 90% rationale in the text.
- "This command generates a MSA file (.aln) for each OG.": This "for" loop is a very fine candidate for parallelization. Please remind the user that they could use a job array for this. Otherwise, they may wait a lot of time staring at the shell.
- "there is much to gain by making phylogenies from multiple gene families": Here, I am strongly opposed to the words "gene families". The whole point of OMA is to identify single-copy (orthologous) genes, not gene families (i.e., including paralogues). Please use instead "from concatenations of multiple genes, rather than from one single gene" (or something similar).
- "because the evolutionary history of a single gene can be misrepresentative of a species evolutionary history": This is said twice in the manuscript (and this is true) but not backed up by specific reasons. Please enumerate a few of them between parentheses.
- In the legend of Figure 3 and 4, please provide the evolutionary model used. I know it is in Table 2, but somewhat hidden in the command line arguments of the software packages.
- "in this case both trees have identical topology" [Figure 4]: This is correct, but this topology is widely different from the two topologies of Figure 3. This shows that phylogenetic artefacts are at play (which is well known for yeasts...) and this might worth mentioning in the sentences about the caveats of phylogenomics. In other words, it is a pity that this use case does not provide a clear phylogenetic solution for the selected genomes.

Is the rationale for developing the new method (or application) clearly explained?

Yes

Is the description of the method technically sound?

Yes

Are the conclusions about the method and its performance adequately supported by the findings presented in the article?

Yes

If any results are presented, are all the source data underlying the results available to ensure full reproducibility?

Yes

Are sufficient details provided to allow replication of the method development and its use by others?

Partly

Reviewer Expertise:

Phylogenomics, comparative genomics, software engineering

I confirm that I have read this submission and believe that I have an appropriate level of expertise to confirm that it is of an acceptable scientific standard, however I have significant reservations, as outlined above.

## References

[1] : Pitfalls in supermatrix phylogenomics. *European Journal of Taxonomy* .2017; 10.5852/ejt.2017.283 10.5852/ejt.2017.283

# Response

**Published:** 2022-02-10

**Authors:** Natasha Glover

**Affiliations:** Department of Computational Biology, Swiss Institute of Bioinformatics, Switzerland

**We thank the reviewer for their very helpful, clear, and pertinent remarks. Each original comment is in italics and our responses are in bold.**

*1. "16 rRNA ribosomal genes" should rather be "small subunit ribosomal RNA (SSU rRNA 16S/18S) gene" (no plural) to encompass the eukaryotic gene as well.*

**Done.**

*2. "see 3 for common pitfalls in phylogenomics": Don't you mean Philippe et al. (2017) "Pitfalls in supermatrix phylogenomics 1" instead?*

**The previous reference addressed most of the same issues as the one the reviewer suggests, but the latter is more recent and comprehensive. We now cite both.**

*3. "OGs can have at most one representative gene per species": You should probably say a word about recently diverged in-paralogs (which do not harm species phylogenies) because exactly single-copy genes barely exist.*

**We have now updated the text to describe how in-paralogs are handled: “If recently diverged in-paralogs are inferred (i.e., co-orthologs), only one of the copies will be selected for the OG. Thus, all members of the OG are still orthologous to each other.”**

*4. "among more than 2300 genomes across the tree of life": You might provide a few numbers here: bacteria, archaea, non-animals, non-plants - or at least recall the rationale for genome inclusion in OMA Browser because it is quite incomplete in terms of species diversity.*

**We added more details on species selection and species breakdown on major clades. We also updated the numbers to be in line with the latest release of OMA: “...provides orthology information among more than 2400 genomes across the tree of life, selected to maximize taxon coverage and users’ needs (Altenhoff *et al.* 2021); to date, there are 1710 Bacteria, 153 Archaea, and 561 Eukaryotes.”**

****

*5. "using species in OMA in addition to other genomes not available in the database": It is not clear if OMA Standalone requires conceptual translations of the sequences (i.e., proteins) or if it can translate DNA sequences on the fly; if it cannot, please specify that one needs protein sequences and not genomes to make use of OMA.*

**We added precision on the input format specification for Protocol 2. Since it needs to be compatible with precomputed OMA data, only protein sequence may be used for this Protocol. We updated the text at the end of the Introduction to say “using species in OMA in addition to other proteomes not available in the database, e.g. a proteome obtained from sequencing a new species. By proteome we mean all protein or nucleic acid sequences of protein-coding genes annotated in a genome.”**

**We also changed the later text in “Combining the added genomes with exported OMA data” to read: “For this procedure, the added genomes data must fulfil certain conditions: Each additional dataset is in the form of a fasta file, containing protein sequences of all coding genes in the corresponding genome. Please note that OMA Standalone can work on nucleic coding sequences when starting from scratch, however for compatibility with the pre-computed OMA data, only protein sequences may be used when combining new and exported data. ”**

*6. "phylogenetic tree inference softwares": I am not a native English speaker, but "software" is uncountable; maybe you mean "software packages"?*

**We corrected this error, following the reviewer's suggestion.**

****

*7. IQtree, RaxML, Phylobayes, Figtree etc: Please try to use the correct mixture of uppercase and lowercase letters in each name across the manuscript: IQ-TREE, RAxML, PhyloBayes, FigTree.*

**We thank the reviewer for pointing out this oversight. Now all the mentioned software names have been corrected.**

****

*8. A better download address for PhyloBayes would be: http://www.atgc-montpellier.fr/phylobayes/.*

**Thanks, corrected.**

****

*9. The interface of OMA Browser has slightly changed since the manuscript has been written. Two details that would need updating: "in the upper left of the home screen" is incorrect [for "Explore"] and "Compute" has now become "Download".*

**We changed the text to be adapted to the latest version of the browser. We updated Figure 1 as well to reflect the new menus and design.**

****

*10. "To speed up the tree inference, set this value to below 1000 genes": One wonders how these <1000 genes would be prioritized and selected; this should be mentioned here.*

**When the number of markers is limited, Orthologous Groups are selected in decreasing order of species representation. This ensures the genes with representatives in most of the selected species will be prioritized. We adapted the text to reflect this.**

*11. "The name of the fasta file is either the species name or the 5-letter UniProt identifier...": This is not specific enough; what about whitespace, dots in strain names, etc? Are they allowed? Similarly, you should better define "other special characters" just below.*

**We agree, this could be clarified. We now changed the text to say:**

**“The added genomes data must fulfill certain conditions:**

**-The name of the fasta file should identify the species clearly and uniquely. The exported genomes from OMA use for example UniProt’s mnemonic five-letter species codes. The filename must end with a “.fa” suffix and must not contain any whitespace characters. The filename without the “.fa” suffix is used as the species name throughout the process and result files.**

**-Each sequence in the fasta file has a clear and unique identifier. We suggest not to use special characters such as brackets, dots, or a pipe character. The reason is that many programs use them for special purposes, e.g. brackets are used in the newick format for tree representation, and the pipe character is often used to separate ids and annotations.”**

****

*12. "...and not keeping other unrelated orthology inferences": This is a bit mysterious. I am aware that OMA can do more than what you demonstrate here, but this sentence should probably be slightly expanded with some examples.*

**We added more details concerning Hierarchical Orthologous Group, which is the main thing we referred to here. We changed the text to say: “If the goal is to only generate a dataset for species phylogeny inference (and not to keep other unrelated orthology inferences, such as Hierarchical Orthologous Groups (Zahn-Zabal *et al.* 2020), which are better representation of individual genes’ evolutionary histories but take time to compute), one can avoid doing computations and generating output files that are not needed by the following:...”**

****

*13. "...use the job-array syntax in the submission script": This is the part where reproducibility is not straightforward. In particular, is there a master OMA instance controlling the parallelization or is this left to the user? From the code snippet provided, it is not clear, albeit the output of the oma-status command suggests that there is some orchestrator freeing the user from this chore. Please explain a bit more.*

**We try to make this more clear by now saying: “OMA Standalone automatically partitions the work chunks in a static and deterministic way among the specified number of workers. Progress of the entire computation can be checked with the OMA Status command (see below).”**

****

*14. "we selected 880 OGs with at least 18 species as a dataset": Considering Figure 4, I understand your point: 90% of 20 species is 18. However, since you start with 18 genomes and add two "new" ones, the re-use of the number "18" is confusing to the user. As the analyses are done, I would not suggest to change this number, but rather to explain directly the 90% rationale in the text.*

**We reformulated a bit to make the reasoning more explicit. We now say: “Once the computation finished, we selected 880 OGs that included at least 90% of the 20 species --18 -- as a dataset to construct a tree.”**

****

*15. "This command generates a MSA file (.aln) for each OG.": This "for" loop is a very fine candidate for parallelization. Please remind the user that they could use a job array for this. Otherwise, they may wait a lot of time staring at the shell.*

**We now add a reminder that using job arrays is a recommended solution with big datasets. We changed the text to say: “This command sequentially generates a MSA file (.aln) for each OG. Depending on the number of OG and species in your dataset, executing it may take a prohibitive amount of time. If it is the case, we recommend using job-arrays to execute the alignments in parallel.”**

****

*16. "there is much to gain by making phylogenies from multiple gene families": Here, I am strongly opposed to the words "gene families". The whole point of OMA is to identify single-copy (orthologous) genes, not gene families (i.e., including paralogues). Please use instead "from concatenations of multiple genes, rather than from one single gene" (or something similar).*

**We agree this phrase is misleading because even if OMA also provides orthology at the level of whole gene families (HOGs) we are using OGs here - single-copy orthologs. We changed the text as suggested by the reviewer.**

****

*17. "because the evolutionary history of a single gene can be misrepresentative of a species evolutionary history": This is said twice in the manuscript (and this is true) but not backed up by specific reasons. Please enumerate a few of them between parentheses.*

**We now enumerate some reasons and link to a reference on the first time this is  mentioned, in the introduction.**

****

*18. In the legend of Figure 3 and 4, please provide the evolutionary model used. I know it is in Table 2, but somewhat hidden in the command line arguments of the software packages.*

**We now provide the evolutionary models used in both figures. The legends now say “Comparison of phylogenetic trees computed by IQ-TREE, using an LG substitution model (left), and RAxML, using an LG substitution model, a discrete Gamma model of rate heterogeneity with 8 categories, and empirical amino-acid frequencies (right)...”**

*19. "in this case both trees have identical topology" [Figure 4]: This is correct, but this topology is widely different from the two topologies of Figure 3. This shows that phylogenetic artefacts are at play (which is well known for yeasts...) and this might worth mentioning in the sentences about the caveats of phylogenomics. In other words, it is a pity that this use case does not provide a clear phylogenetic solution for the selected genomes.*

**We thank the reviewer for pointing this out. It is true that there are some differences between the species tree obtained from the two protocols and that it must be addressed. The difference corresponds to bipartitions that are not well supported in either species tree but this was not easily apparent in the first version of the manuscript.**

**We made two major change to address this comment:**

- **We changed Figures 3 and 4 to show the least statistically bipartitions in the species tree.**
- **We added a paragraph in the discussion pointing out the difference between the species tree and discussing the importance of accounting for statistical support when constructing a species phylogeny.**

# Peer review: Reviewer response for version 1

**DOI:** 10.5256/f1000research.26251.r84386

**Published:** 2021-05-25

**Authors:** Jianbo Xie

**Affiliations:** 1 National Engineering Laboratory for Tree Breeding, College of Biological Sciences and Technology, Beijing Forestry University, Beijing, China

The manuscript provides useful tools to make use of OMA Orthologous Groups to infer a phylogenetic species tree. This manuscript is an interesting study, but I found some shortcomings that the authors should be improved before the manuscript can be considered for indexing. Some marker gene databases, such as Qiyun Zhu *et al. *(2019) ^1^, have been published - could you integrate the database into your tool? Or describe the advantage of your tool?

**Minor:**

      1. The introduction section should include some similar tools such as PGAP, Orthomcl. 

      2. Please discuss the advantage of OMA compared with other tools.

      3. Could you integrate the steps of analyses to one steps with multiple parameters?

Is the rationale for developing the new method (or application) clearly explained?

Yes

Is the description of the method technically sound?

Yes

Are the conclusions about the method and its performance adequately supported by the findings presented in the article?

Yes

If any results are presented, are all the source data underlying the results available to ensure full reproducibility?

Yes

Are sufficient details provided to allow replication of the method development and its use by others?

Yes

Reviewer Expertise:

Genetics; evolution

I confirm that I have read this submission and believe that I have an appropriate level of expertise to confirm that it is of an acceptable scientific standard, however I have significant reservations, as outlined above.

## References

[1] : Phylogenomics of 10,575 genomes reveals evolutionary proximity between domains Bacteria and Archaea. *Nature Communications* .2019;10(1) : 10.1038/s41467-019-13443-4 10.1038/s41467-019-13443-4 PMC688931231792218

# Response

**Published:** 2022-02-10

**Authors:** Natasha Glover

**Affiliations:** Department of Computational Biology, Swiss Institute of Bioinformatics, Switzerland

**We thank the reviewer for their comments, which are listed below (in italics) with our responses (in bold):**

*The manuscript provides useful tools to make use of OMA Orthologous Groups to infer a phylogenetic species tree. This manuscript is an interesting study, but I found some shortcomings that the authors should be improved before the manuscript can be considered for indexing. Some marker gene databases, such as Qiyun Zhu et al. (2019) 1, have been published - could you integrate the database into your tool? Or describe the advantage of your tool?*

**We now mention the marker gene database as the reviewer suggests. This tutorial’s aim is mainly to indicate how to use OMA for inferring marker genes from orthology relationships and then construct a species tree. We do not think it is desirable to integrate other tools into our protocol.**

**One of the clear advantages in using OMA is a clear procedure which can work for any subset of species.**

**We now make it more clear in the text, and state: “It is sometimes possible to rely on existing marker genes used in large-scale studies (for example (Zhu *et al*. 2019)), but they are generally available only for a subset of species and do not include newly sequenced species.”**

*Minor:*

*1. The introduction section should include some similar tools such as PGAP, Orthomcl.*

**In the introduction, we chose to mention only OMA and BUSCO, because they are to our knowledge the only maintained software that provides single-copy orthologs (does not include any paralogs). This is a prerequisite to use the supermatrix approach for species tree inference.**

****

*2. Please discuss the advantage of OMA compared with other tools.*

**We added a line indicating the main advantage of OMA compared to other methods: it is possible to use a precomputed dataset, and also to mix it with local genomic data, which makes it usable in numerous contexts. We now state:**

**“This type of Orthologous Group is provided by only a few orthology databases such as BUSCO (8) and  Orthologous MAtrix (OMA) (Altenhoff *et al*. 2019; Altenhoff *et al*. 2018) and, to our knowledge, only OMA allows for use of both precomputed and user-computed OGs.”**

****

*3. Could you integrate the steps of analyses to one steps with multiple parameters?*

**As the scope of the paper is to provide a tutorial, we do not plan on integrating the steps into only one command line script.**

**One of the main reasons to not integrate it into a single command line is to leave more choice to the user regarding the non-OMA tools they may use, as well as their parameters. Given the high number of possible parameters at any step, it would make designing and using such a script highly complicated.**

**A second reason is that such an implementation will need to make technical choices (e.g regarding parallelization) which would make it incompatible with some computing infrastructures.**
